# T Cell Subset and Stimulation Strength-Dependent Modulation of T Cell Activation by Kv1.3 Blockers

**DOI:** 10.1371/journal.pone.0170102

**Published:** 2017-01-20

**Authors:** Wai-Ping Fung-Leung, Wilson Edwards, Yi Liu, Karen Ngo, Julianty Angsana, Glenda Castro, Nancy Wu, Xuejun Liu, Ronald V. Swanson, Alan D. Wickenden

**Affiliations:** Janssen Research & Development, L.L.C., San Diego, California, United States of America; University of Alberta, CANADA

## Abstract

Kv1.3 is a voltage-gated potassium channel expressed on T cells that plays an important role in T cell activation. Previous studies have shown that blocking Kv1.3 channels in human T cells during activation results in reduced calcium entry, cytokine production, and proliferation. The aim of the present study was to further explore the effects of Kv1.3 blockers on the response of different human T cell subsets under various stimulation conditions. Our studies show that, unlike the immune suppressor cyclosporine A, the inhibitory effect of Kv1.3 blockers was partial and stimulation strength dependent, with reduced inhibitory efficacy on T cells under strengthened anti-CD3/CD28 stimulations. T cell responses to allergens including house dust mites and ragweed were partially reduced by Kv1.3 blockers. The effect of Kv1.3 inhibition was dependent on T cell subsets, with stronger effects on CCR7^-^ effector memory compared to CCR7^+^ central memory CD4 T cells. Calcium entry studies also revealed a population of CD4 T cells resistant to Kv1.3 blockade. Activation of CD4 T cells was accompanied with an increase in Kv1.3 currents but Kv1.3 transcripts were found to be reduced, suggesting a posttranscriptional mechanism in the regulation of Kv1.3 activities. In summary, Kv1.3 blockers inhibit T cell activation in a manner that is highly dependent on the T cell identity and stimulation strength, These findings suggest that Kv1.3 blockers inhibit T cells in a unique, conditional manner, further refining our understanding of the therapeutic potential of Kv1.3 blockers.

## Introduction

Kv1.3 is a voltage-gated potassium channel (Kv) which opens in response to membrane depolarization [1]. Functional Kv1.3 is comprised of a homotetramer of pore forming alpha subunits and membrane depolarization is sensed by positively charged arginine residues in the fourth transmembrane region of each subunit [2]. Kv1.3 has been suggested to play a role in T cell activation [1, 3–8]. T cells are activated through TCR (T cell receptor) engagement with specific antigenic peptides presented by self MHC molecules on antigen presenting cells [9]. Multiple signaling cascades including MAPK, NF-kB and NFAT pathways are activated by the TCR complex [10–12]. NFAT pathway is a calcium dependent signaling pathway that requires a sustained calcium flux to activate the phosphatase calcineurin and the downstream transcription factor NFAT for induction of gene expression [13–15]. Calcium mobilization in T cells is mediated by the store-operated calcium channel known as calcium release activated calcium (CRAC) channel, which is recruited to the immunological synapse upon TCR engagement [16]. Kv1.3 is also recruited to the immunological synapse and is thought to be required for sustaining the CRAC mediated calcium flux [3, 7, 17–19].

Peptides isolated from the venoms of various creatures have proven valuable as tools to explore the functional role of Kv1.3 channels. ShK peptide toxin from the Caribbean sea anemone Stichodactyla helianthus, and members of the α-KTx3 scorpion toxin family, such as OsK1 from the venom of the Central Asian scorpion Orthochirus scrobiculosus and OdK2 from the Iranian scorpion Odonthobuthus doriae, are all potent blockers of Kv1.3 [5, 20–23]. Engineered variants of ShK, OsK1 and OdK2 that potently and selectively inhibit Kv1.3 have also been identified [24, 25]. Recently we reported an engineered Kv261 peptide with sequence derived from OsK1 and OdK2 [24]. We demonstrated that Kv261 and its human albumin fusion protein Kv261-HSA-34 are potent and selective Kv1.3 blockers [24].

Numerous studies have shown that Kv1.3 blockers inhibit T cell activation [1, 3–8]. Kv1.3 blockers have also been reported to be efficacious in animal models of T cell mediated delayed-type hypersensitivity (DTH), experimental autoimmune encephalomyelitis, arthritis, autoimmune diabetes, transplantation, allergic dermatitis and psoriasis [6, 7, 25–33], raising the possibility that Kv1.3 blockers may have the potential for treatment of human autoimmune diseases. However, our understanding of the effects of Kv1.3 blockers on T cell function is still limited. The inhibition of T cells by Kv1.3 blockers often appears to be less robust than clinically effective immune suppressors, and their effects seem to vary considerably among different species and human donors [4, 5]. The impact of Kv1.3 blockade also seems to be influenced by T cell identity [4, 6, 8]. More information on the profile of Kv1.3 blocker activities across various T cell subsets under different stimulation conditions is needed to better understand the therapeutic potential of this emerging drug class.

In this report we compared the effects of Kv1.3 blockers with the immune suppressor, cyclosporine A, on proliferation, cytokine production and calcium flux in purified human primary T cell subsets under different stimulation conditions. Our studies confirm and extend previous observations and further suggest that inhibition of T cells by Kv1.3 blockers varies in a complex, multifactorial manner, dependent on the nature of the stimulus, the T cell response and the T cell identity.

## Materials and Methods

### Materials

The Kv1.3 channel blockers used in this report were OsK1 peptide (Alomone Labs), ShK peptide (Bachem), Kv261 peptide (Janssen R&D), and Kv261-HSA-34 fusion protein (Janssen R&D). Cyclosporine A (Alexis) and BTP2 (Calbiochem) were used as control inhibitors. Human blood samples were provided by the Scripps Research Institute. The study protocol on human blood samples was submitted by Janssen R&D and approved by the Scripps Research Institute IRB (Institutional Review Board). Blood donors have given written consents to participate in the studies with the procedure approved and records kept by the Scripps Research Institute IRB.

### T cell purification

Human PBMC were purified from donor blood by Ficoll-Paque (GE Healthcare) density centrifugation. CD4 or CD8 T cells were purified from human PBMC by negative selection using human CD4 or CD8 T cell isolation kit (Miltenyi Biotec) respectively. Naïve or memory CD4 T cells were isolated from CD4 T cells by negative selection using human naïve or memory CD4 T cell isolation kit (Miltenyi Biotec) respectively. Central and effector memory CD4 T cells were separated from memory CD4 T cells using biotinylated anti-CCR7 (BD Biosciences) and streptavidin coated magnetic beads (Miltenyie Biotec). Cells after treatment with antibodies and magnetic beads were purified with autoMACS (Miltenyi Biotec).

### Quantitative RTPCR gene expression

RNA was extracted from different human T cell samples using RNeasy Plus mini kit (Qiagen). RNA was converted to cDNA by reverse transcription using the Gene-amp Gold RNA PCR kit (Thermo Fisher). Quantitative PCR assays were set up using primers specific for Kv1.3 (HS00704943_s1, Thermo Fisher) and GAPDH (Hs02758991_g1, Thermo Fisher) and TaqMan Universal PCR Master Mix (Thermo Fisher). PCR reactions were performed in Applied Biosystems® 7500 Real-Time PCR System and reaction conditions were set at 50°C for 2 minutes, 95°C for 10 minutes, 40 cycles of 95°C for 15 seconds, followed by 60°C for 1 minute. Expression relative to GAPDH was calculated using 2ΔCt.

### Microarray gene expression profile

Global gene expression profiles in 128 normal human tissues (sourced from Biochain, Ambion and Stratagene) were performed using Affymetrix GeneChip HT HG-U133+ PM arrays. Gene expression was first measured at the probe set level, using the RMA (Robust Multiarray Average) methodology, followed by quantile normalization. Kv1.3 mRNA expression was measured and compared among different tissues.

### Patch–clamp electrophysiology

The potency of OsK1 peptide against voltage-gated currents in human primary CD4 T cells and CHO cells stably transfected with recombinant human Kv1.3 was measured in a manual patch clamp assay, as previously described [24]. Briefly, T cells or transfected CHO cells were plated onto glass coverslips in a bath on the stage of an inverted microscope and perfused (approximately 1ml/min) with extracellular solution of the following composition: 137mM NaCl, 2mM CaCl_2_, 5.4mM KCl, 1mM MgCl_2_, 5mM glucose, and 10mM HEPES, 0.1% bovine serum albumin, pH 7.4. Pipettes were filled with an intracellular solution of the following composition: 40mM KCl, 100mM KF, 2mM MgCl_2_, 10mM EGTA, 10mM HEPES, pH 7.3 to 7.4, and had a resistance of 2 to 4 MΩ. All recordings were made at room temperature (22–24°C) using a Multiclamp 700A amplifier and pClamp 9 software (Axon Instruments). Outward potassium currents were measured using the whole-cell configuration of the patch-clamp technique. The protocol consisted of a 200ms step to a test potential of +40mV, followed by a 100ms repolarization to -40mV prior to returning to the holding potential of –80mV. In a small number of experiments with recombinant Kv1.3, test potentials of either +20mV or +30mV were used (rather than +40mV) to limit the amplitude of the outward potassium current. Current records were acquired at 2–5 KHz and filtered at 1–2 KHz. Uncompensated series resistance was typically < 10MΩ and 80% series resistance compensation was routinely applied. Currents were elicited once every 20s and were allowed to stabilize for 5–10 minutes prior to recording. Blockers were applied using an SF-77B Fast-Step Perfusion device (Warner Instruments) and 1–4 concentrations of blockers were tested per cell.

The number of Kv1.3 channels expressed by purified human T cells was measured using a PatchXpress 7000A Automated Patch Clamp System (Molecular Devices). Extra- and intracellular solutions were the same as for manual patch clamp studies. Outward potassium currents were elicited with 100ms steps to a voltage in the range of +20 to +40mV from a holding potential of –80mV in the absence and presence of 100nM ShK. Channel numbers were calculated from the amplitude of the ShK-sensitive current, assuming a single channel conductance for Kv1.3 of 12pS [5].

CD4 T cells were purified from human PBMC as described in the T cell purification section. Activated T cells were prepared by stimulating cells with anti-CD3/anti-CD28 coated Dynabeads (Life Technologies) at 1:1 bead/cell ratio for 2 days or at other time points as indicated. Expression levels of CD25 and CD69 on activated T cells were monitored in flow cytometer after staining with fluorescent dye conjugated specific antibodies (eBiosciences). House dust mite-specific T cells were generated by stimulating CD4 T cells with house dust mite extract (Greer) in the presence of mitomycin C (Sigma-Aldrich) treated autologous PBMC. CD4 T cells were purified from day 11 cultures using the CD4 T cell purification kit (Miltenyi Biotec). Purified antigen specific CD4 T cells were activated for 2 days with 2μg/ml concanavalin A (Sigma-Aldrich) before testing in PatchXpress assays.

### T cell activation assays

T cells were cultured in 96-well plates (Thermo Scientific) with 200μl medium/well. Culture medium was RPMI-1640 medium supplemented with 2% human A/B serum (Valley Biomedical Prod & Srv Inc.), 2 mM glutamine, 1mM sodium pyruvate, 10mM HEPES, 1mM MEM nonessential amino acid solution, and 100 U/ml each of penicillin G and streptomycin (all culture reagents from Life Technologies except for human serum). T cells were pre-treated with Kv1.3 blockers and other inhibitors for 30 minutes before stimulation. All assays were conducted with samples in duplicates or triplicates.

For T cell pan-activation with beads coated with anti-CD3 and anti-CD28 (eBioscience), T cells at 2 x 10^5^ cells/well in 96-well flat-bottom plates were stimulated overnight with anti-CD3 and anti-CD28 coated beads (Miltenyi Biotec) at 2:1 bead/cell ratio or as indicated. For T cell pan-activation with anti-CD3, T cells at 5 x 10^4^ cells/well in 96-well U-bottom plates were stimulated for 2 days with 1ng/ml of anti-CD3 in the presence of mitomycin C treated autologous PBMC at 1:1 T cell/PBMC ratio. Antigen-specific activation of CD4 T cells was set up in the same way as the anti-CD3 assay except that 10μg/ml specific antigens were used and stimulations were for 4 days. Antigens used in T cell stimulation include house dust mite (Dermatophagoides pteronyssinus) extract, ragweed extract (Greer), and purified tetanus toxoid (University of Massachusetts Biologic). Autologous PBMC used as antigen presenting cells in T cell activation assays were pre-treated with 50 μg/ml mitomycin C for 20 minutes followed by rinsing with an excess amount of PBS, and finally taken up in culture medium for assay set up.

### Thapsigargin stimulation assays

Heparinized human blood was diluted with an equal volume of IMDM medium (Life Technologies) supplemented with 2% normal human A/B serum and plated at 200μl/well in 96-well plates for thapsigargin (Alomone) stimulation. CD4 T cells were plated at 5 x 10^5^ cells/well in 96-well plates with 200μl medium/well. Blood or T cell samples were pre-treated with blockers or inhibitors for 30 minutes prior to stimulation with thapsigargin at 10μM or other concentrations as indicated for 20 hours. Cytokines in culture supernatants were measured as described in cytokine detection section.

### Proliferation measurement

Proliferation of T cells was measured by overnight pulsing with 1μCi/well of ^3^H-thymidine (Perkin Elmer). Cells with incorporated radioactive thymidine were harvested onto glass fiber filter plates (Perkin Elmer). Filter plates were soaked with scintillant (Perkin Elmer) and radioactivity was counted using a Topcount (Packard).

### Cytokine measurement

Cytokines in supernatants collected from blood or T cell cultures were measured with ELISA or electrochemiluminescent assays. IL-2 was measured by ELISA using the human IL-2 Quantikine Kit (R&D Systems). Other cytokines were detected using multiplex human cytokine electro-chemiluminescent kit (Meso Scale Discovery).

### Calcium flux measurement

T cells were loaded with 3μM each of Fluo4 and Fura red (Molecular Probes, Invitrogen) for 45 minutes in the dark at 37°C in cell loading medium (DMEM medium with 10mM HEPES and 10% fetal calf serum) in the presence of 0.01% pluronic acid (Molecular Probes). Dye-loaded cells were rinsed twice with loading medium, incubated with 10μg/ml mouse anti-CD3 (BD Biosciences) and 2.5μg/ml mouse anti-CD28 (BD Biosciences) for 30 minutes at room temperature, and then rinsed again with loading medium to be ready for calcium flux studies. Cells were acquired with FACS LSRII and cell activation was initiated by addition of goat anti-mouse antibody (Jackson ImmunoResearch) to final 10μg/ml. CD4 T cells were also stimulated with 1μM thapsigargin. Kinetics of calcium flux in activated cells were monitored by reading the calcium dependent Fluo4 emission signals at 530nm compared to non-specific Fura red signals at 685nm. Calcium flux was calculated as ratio of Fluo4 to Fura red emission signals.

### Data analysis and statistics

Concentration- response curves and IC_50_ values were generated from non-linear regression. P values were generated from Students T test or 2-way ANOVA as indicated. All analyses were performed using PRISM program (GraphPad).

## Results

### Stimulation strength-dependent efficacy of Kv1.3 blockers on human T cells

**Fig 1:**
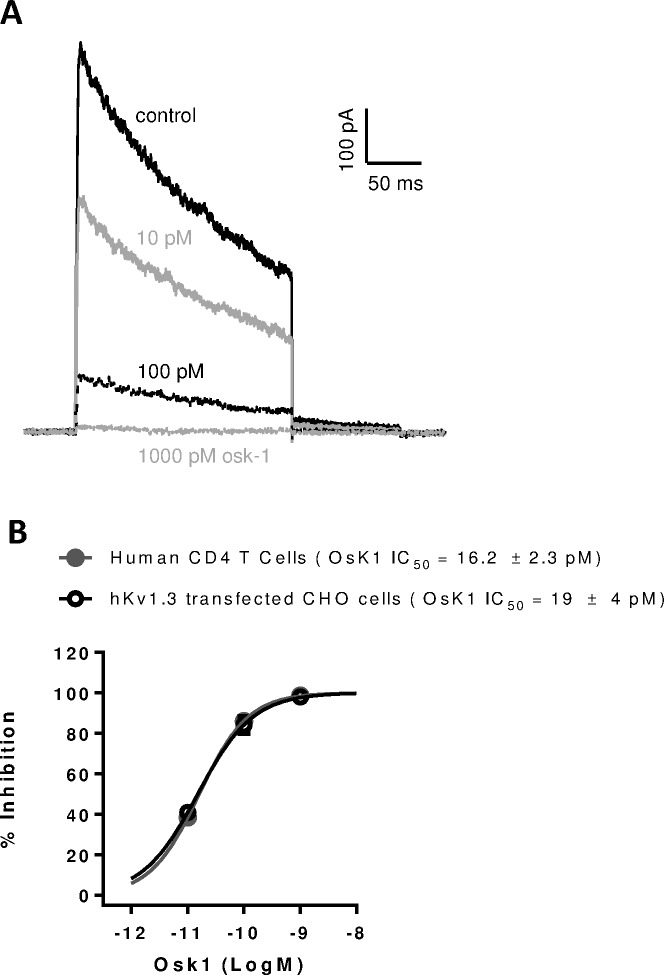
OsK1 peptide inhibited voltage-gated Kv1.3 currents in human CD4 T cells and CHO cells transfected with recombinant human Kv1.3. OsK1 peptide inhibited human Kv1.3 mediated whole cell currents in a concentration dependent manner in human CD4 T cells (A). Potency of OsK1 peptide on Kv1.3 currents in purified human CD4 T cells and Kv1.3 transfected CHO cells were determined and IC_50_ curves were shown (B). Whole cell voltage-gated currents were measured in manual patch clamp assays. All recordings were made at room temperature using Multiclamp 700A amplifier and pClamp 9 software as described in Methods and Materials.

To investigate the role of Kv1.3 in T cell activation, we tested different Kv1.3 blockers in our T cell assays. The potencies of Kv1.3 blockers used in our studies were determined in patch clamp assays against recombinant human Kv1.3 channels in stably transfected in CHO cells which do not have endogenous Kv currents [34] (Table 1). The IC_50_ values of different Kv1.3 blockers determined on CHO transfected cells were similar to those published in other studies [35, 36]. OsK1 peptide was used in the majority of our experiments and it showed inhibitory effects on endogenous Kv1.3 in human primary CD4 T cells in a concentration dependent manner (Fig 1A). OsK1 potency on endogenous Kv1.3 in human CD4 T cells (IC_50_ 16.2±2.3pM) was found to be comparable to its potency on recombinant Kv1.3 in CHO cells (IC_50_ 19±4pM) (Fig 1B).

**Table 1:** Potency of different Kv1.3 blockers on human Kv1.3 channels on transfected CHO cells.

Kv1.3 Blocker	IC_50_ ± SEM (pM)
ShK	24 ± 12
OsK1	19 ± 4
OdK2	100 ± 10
Kv261	19 ± 3
Kv261-HSA-34	1300 ± 370

**Fig 2:**
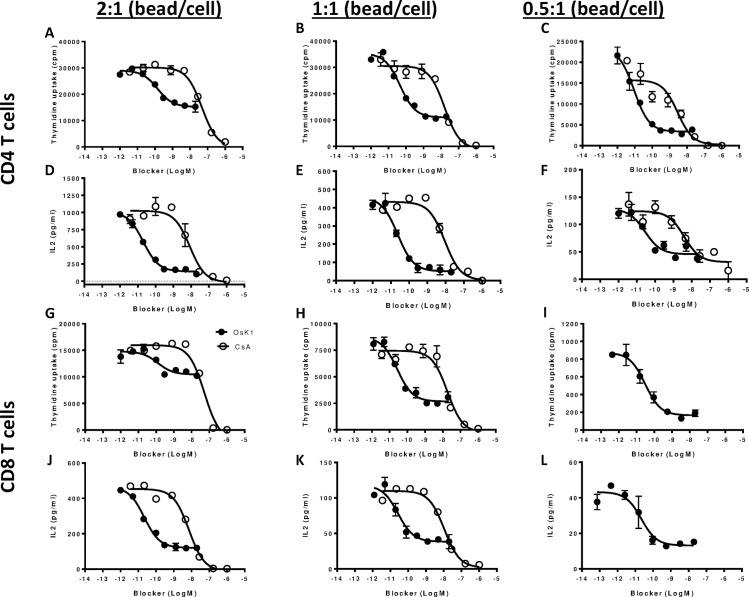
Efficacy of Kv1.3 blocker OsK1peptide in inhibiting T cell activation is partial and dependent on stimulation strength. Effect of OsK1 peptide (filled circles) or cyclosporine A (open circles) on proliferation or IL-2 production of human primary CD4 (A—F) and CD8 T cells (G—L) activated with anti-CD3/CD28 coated beads under strong stimulation (2:1 bead:cell ratio; left panels), intermediate stimulation (1:1 bead:cell ratio; middle panels), or weak stimulation (1:2 bead:cell ratio; right panels) conditions. Cyclosporine A was not tested in CD8 T cells with weak stimulation due to insufficient purified T cells. The assays were performed in 4 independent experiments with samples in duplicates. Data from one representative experiment were presented.

To monitor the effect of Kv1.3 blockers on T cell activation, CD4 and CD8 T cells were purified from human blood and stimulated with anti-CD3/anti-CD28 coated beads in the presence of OsK1 peptide. The calcineurin inhibitor, cyclosporine A, was used as a positive control. OsK1 peptide potently inhibited proliferation of CD4 and CD8 T cells at bead/T cell stimulation ratio of 1:1 (Fig 2B, H; Table 2), but inhibition was only partial, even at maximally effective blocker concentrations. The blocking efficacy of OsK1 peptide was further investigated in T cells stimulated with different stringencies by adjusting the ratio of anti-CD3/anti-CD28 coated beads and T cells. As shown in Fig 2, both CD4 and CD8 T cells showed a corresponding enhanced proliferation and IL-2 production when the ratio of bead/T cell in cultures was increased from 0.5:1 to 1:1 and 2:1. OsK1 peptide blocked CD4 T cell proliferation with efficacies of 80%, 66% and 44% inhibition when stimulated at bead/T cell ratio of 1:2, 1:1 and 2:1 respectively (Fig 2C, 2B and 2A). Reduced OsK1 peptide inhibitory efficacy with increased T cell stimulation strength was also observed in CD8 T cell proliferation, where maximal efficacy was 82%, 70% and 26% at 1:2, 1:1 and 2:1 bead/T cell stimulation ratios, respectively (Fig 2I, 2H and 2G). There also appeared to be a trend of reduced OsK1 peptide potency on the proliferation of CD4 or CD8 T cells under the most stringent stimulation condition (Fig 2, Table 2). Partial efficacy of OsK1was also observed on inhibition of IL-2 secretion, but in contrast to the effects on T cell proliferation, its potency and efficacy on IL-2 production was not significantly affected by variation in T cell stimulation strength (Fig 2D–2F and 2J–2L). The blocking profile of OsK1 peptide was distinct from cyclosporine A, as high concentrations of cyclosporine A blocked all T cell activation close to 100% in all T cell readouts.

**Table 2:** Potency and efficacy of Kv1.3 blockers on human T cells under different stimulation conditions.

Cell Type	Stimulus		Kv1.3 Blockers	Proliferation		IL-2	
				Efficacy (%Inhibition)	Potency (IC_50_, pM)	Efficacy (%Inhibition)	Potency (IC_50_, pM)
CD8 T cell	Anti-CD3/CD28 coated beads	Bead/cell 2:1	OsK1	39.3 ± 1.8	125.6 ± 0.4	77.4 ± 1.1	21.9 ± 1.9
		Bead/cell 1:1	OsK1	68.8 ± 0.2	27.3 ± 4.0	63.3 ± 9.4	27.0 ± 9.7
		Bead/cell 1:2	OsK1	85.6 ± 0.1	30.7 ± 13.3	69.8 ± 1.1	21.7 ± 14.6
CD4 T cell	Anti-CD3/CD28 coated beads	Bead/cell 2:1	OsK1	36.0 ± 8.8	139.7 ± 1.0	85.6 ± 2.4	20.6 ± 4.5
		Bead/cell 1:1	OsK1	62.7 ± 3.7	45.9 ± 9.2	90.0 ± 3.7	23.8 ± 5.0
		Bead/cell 1:2	OsK1	82.2 ± 0.1	15.3 ± 5.0	73.3 ± 1.4	27.9 ± 6.1
CD4 T cell	Thapsigargin		OsK1	35.5 ± 3.7	40.0 ± 2.8	71.1 ± 2.4	6.8 ± 3.1
			Kv261	32.0 ± 3.9	48.4 ± 1.7	74.6 ± 1.5	7.0 ± 0.6
CD4 Tcm cell	Anti-CD3/PBMC		OsK1	38.8 ± 2.8	95.6 ± 41.8	BLD*	BLD*
CD4 Tem cell	Anti-CD3/PBMC		OsK1	74.5 ± 4.3	51.1 ± 5.7	BLD*	BLD*
CD4 naïve cell	Anti-CD3/PBMC		OsK1	62.7 ± 7.7	776.5 ± 179.5	BLD*	BLD*
House dust mite-specific T cell	House dust mite extract		OsK1	58.9 ± 4.3	41.6 ± 26.1	ND**	ND**
			Kv261	37.8 ± 9.7	14.1 ± 28.7	ND**	ND**
			Kv261-HSA-34	72.6 ± 3.6	3397 ± 1038	ND**	ND**

* BLD: Below detection limit.

** ND: not determined.

**Fig 3:**
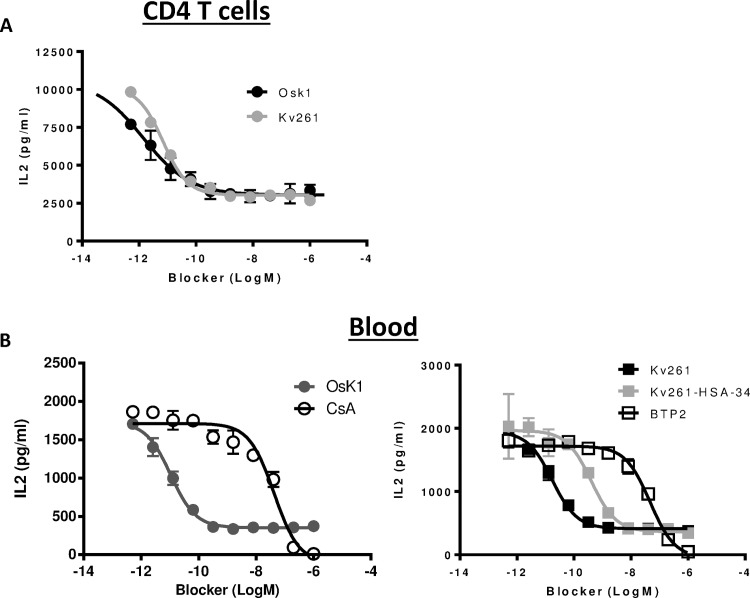
Effect of Kv1.3 blockers effect on cytokine production from thapsigargin stimulated human blood and purified CD4 T cells. Effects of OsK1 and Kv261 on IL-2 production of thapsigargin stimulated CD4 T cells (A) were shown. OsK1, Kv261 peptide and Kv261-HSA-34 were tested in thapsigargin stimulated blood (B) from healthy donors and IL-2 was measured. BTP2 and cyclosporine A were used as reference compounds. The assays were performed twice in CD4 T cells and 3 times in human blood with samples in duplicates. Data from representative experiments were presented.

Given that Kv1.3 blockers are thought to inhibit T cell activation by reducing calcium signaling, we sought to determine if the partial efficacy of Kv1.3 blockers was due to incomplete suppression of calcium signaling or involvement of other calcium independent signaling pathways mediated by TCR. To address this question, we stimulated T cells or blood samples with thapsigargin rather than anti-CD3/anti-CD28. Thapsigargin is a potent inhibitor of sarcoendoplasmic reticulum calcium-ATPase (SERCA) and its effects are mediated exclusively through induction of calcium flux as a result of intracellular calcium store depletion [37]. OsK1 inhibited IL-2 production from thapsigargin-induced CD4 T cells and blood with IC_50_ values corresponding with its potency on Kv1.3 currents (Fig 3A and 3B; Table 2). Similar to findings with anti-CD3/anti-CD28 stimulation, OsK1 only partially inhibited thapsigargin-induced IL-2 secretion. This partial efficacy was also observed on IL-2 release from thapsigargin stimulated whole blood treated with other Kv1.3 blockers Kv261 peptides and Kv261-HSA-34 fusion protein (Fig 3B, Table 2). The result suggests that the observed partial efficacy was a general feature of Kv1.3 blockers and not a compound or assay specific phenomenon. In contrast to Kv1.3 blockers, cyclosporine A and the CRAC channel-blocker BTP2 [38] were fully efficacious against IL-2 secretion from thapsigargin stimulated whole blood.

**Fig 4:**
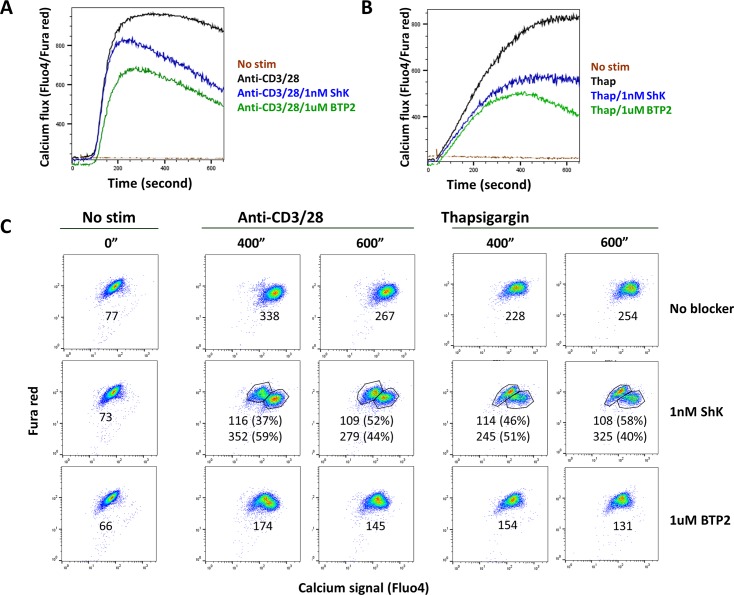
Effect of Kv1.3 blocker on calcium flux in T cells. ShK peptide (1nM) reduced calcium flux induced by either anti-CD3/CD28 (A) or thapsigargin (B) in human CD4 T cells. Kinetics of calcium signal change in different samples are presented as curves over time (A, B). CD4 T cell populations with different levels of calcium signals are presented in dot plots and their Fluo4 intensities in geometric means as well as population percentages are indicated (C). The assays were repeated in 3 independent experiments and data from one representative experiment were presented.

To measure the effects of Kv1.3 blockers on calcium flux, human primary CD4 T cells were purified from blood and loaded with the calcium sensitive and insensitive dyes Fluo4 and Fura red respectively. The dye loaded T cells were then activated with either anti-CD3 and anti-CD28 coupling antibodies, or with the calcium store depleting agent thapsigargin. Calcium flux in CD4 T cells was monitored with flow cytometry as described in Materials and Methods. Addition of ShK peptide at 1nM, or BTP2 at 1μM significantly reduced the calcium flux levels monitored for 10 minutes after activation (Fig 4A and 4B). ShK peptide showed only partial inhibition of calcium flux initiated by coupling of CD3 and CD28 or by thapsigargin (Fig 4A and 4B). We also noted a population of T cells that was able to mount normal calcium flux in the presence of ShK peptide (Fig 4C), whereas BTP2 reduced calcium flux in all activated T cells. The findings with ShK peptide on calcium flux of activated T cells were also confirmed with another Kv1.3 blocker OsK1 peptide (S1 Fig).

### Differential efficacy of Kv1.3 blockers on human CD4 T cell subsets

**Fig 5:**
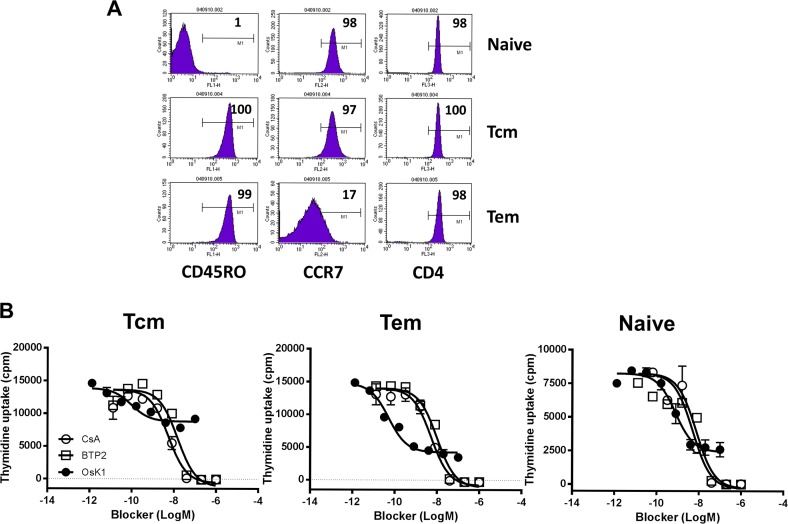
Effect of OsK1 on human CD4 T cell subsets. Human CD45RO^-^ CCR7^+^ naïve T cells, CD45RO^+^ CCR7^+^ central memory (Tcm) and CD45RO^+^ CCR7^-^ effector memory (Tem) T cells were purified from human blood (A). OsK1 peptide blocked proliferation of the purified human CD4 T cell subsets induced by 2 day stimulation with anti-CD3 in the presence of PBMC (B). BTP2 and cyclosporine A were used as reference compounds in T cell assays. The assays were performed in 8 similar experiments with samples in duplicates. Data from one representative experiment were presented.

Kv1.3 blockers have been suggested to block preferentially the activities of effector memory T cells [4, 6–8], however direct, side-by-side comparison of blocker effects on purified primary T cell subsets has not been fully explored. In order to further understand the activity profile of Kv1.3 blockers on T cells, we compared the effects of Kv1.3 blockers on primary naïve, central memory and effector memory CD4 T cells. Human CD4 T cell subsets, namely CD45RA^+^ CCR7^+^ naïve T cells, CD45RO^+^ CCR7^+^ central memory and CD45RO^+^ CCR7^-^ effector memory T cells, were isolated from human blood with high purities of 98%, 97% and 83% respectively (Fig 5A). Purified T cells were activated with anti-CD3 in the presence of mitomycin C treated PBMC and the effects of Kv1.3 blockers on T cell proliferation were monitored and compared to cyclosporine A and BTP2. OsK1 peptide showed a better inhibitory efficacy on naïve (62%) and effector memory CD4 T cells (74%) compared to central memory CD4 T cells (39%) (Fig 5B, Table 2). In contrast, cyclosporine A and BTP2 achieved complete inhibition on different T cell subsets at their maximal efficacious concentrations. Production of IL-2 by T cells in this assay format was below detection levels for evaluation of Kv1.3 blocker effect.

### Expression of Kv1.3 in T cell subsets

**Fig 6:**
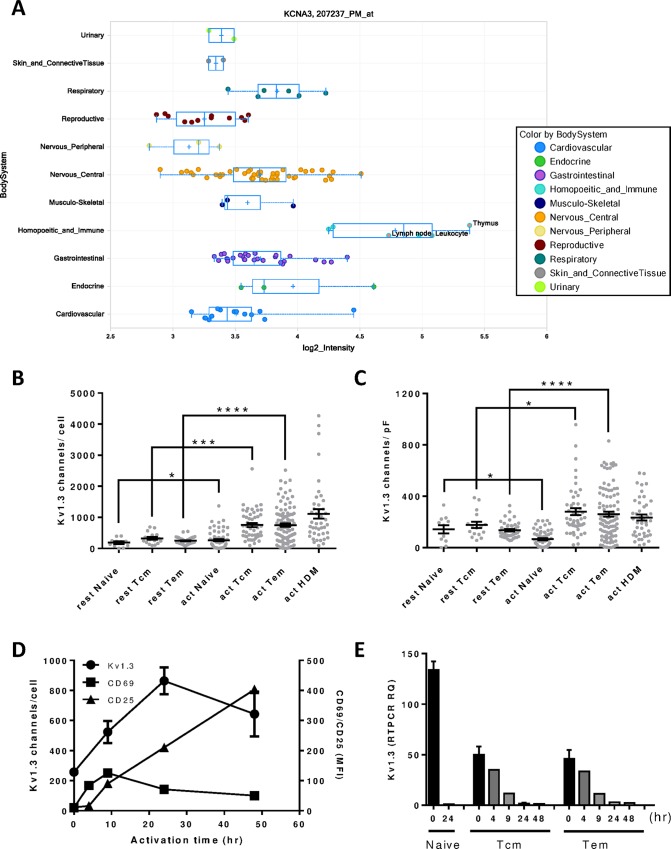
Kv1.3 expression in human tissues and T cells. Kv1.3 mRNA expression in different human tissues was profiled in Affymetrix microarrays (A). Cell surface Kv1.3 channels were measured with patch express assays on activated or resting human CD4 T cell subsets including naïve, central memory (Tcm) and effector memory (Tem) T cells, as well as antigen-specific T cells from house dust mite (HDM) stimulation. Data shown are either channels/cell (B) or number of channels normalized to cell surface area (channels/pF) (C). Time course of Kv1.3 channel activity and expression of the activation markers CD25 and CD69 are shown on effector memory CD4 T cells activated with anti-CD3/CD28 coated beads (D). Expression of Kv1.3 mRNA levels in naïve, Tcm and Tem CD4 T cells at different time points post activation with anti-CD3/CD28 coated beads are presented (E). Patch express measurement of Kv1.3 channels was performed on T cell subsets purified from 6 healthy blood donors. Statistical significance on the difference in Kv1.3 channel levels between activated and resting T cell subsets was analyzed by 2-tailed Student’s t test and P values were determined and indicated as *P < 0.05, ***P < 0.001, ****P < 0.0001.

Kv1.3 mRNA expression was reported in T cell lines and has not been studied in primary T cells [26, 39]. To characterize Kv1.3 expression in human tissues and primary T cells, we profiled Kv1.3 expression transcripts in different human tissues in microarray studies and showed higher expression levels in tissues of the hematopoietic system including bone marrow, lymph nodes, thymus and blood (Fig 6A). These results further suggest the potential role of Kv1.3 in immune cell functions. Differences in channel expression have been suggested as the basis for the differential effect of Kv1.3 blockers on T cell subsets [5, 7, 32]. The number of functional Kv1.3 channels in human T cells subsets including naïve, central memory and effector memory CD4 T cells was studied in patch clamp experiments by measuring the amplitude of the ShK-sensitive, voltage activated outward currents as described in Materials and Methods. In all T cell subsets, the absolute number of Kv1.3 channels was higher in activated compared to resting T cells (Fig 6B, Table 3). This increase was partly due to the increase in cell size that occurs following activation, since differences were smaller when normalized to cell capacitance and presented as channel density (Fig 6C, Table 3). However, there was no significant difference in channel number or density between activated effector memory and activated central memory cells, suggesting Kv1.3 expression alone could not explain differences in the sensitivity to Kv1.3 blockers in these subsets. House dust mite Dp-specific T cells after several rounds of antigen stimulation also demonstrated high levels of Kv1.3 expression (~1000 channels per cell) comparable to the levels on effector and central memory cells (Fig 6B and 6C, Table 3).

**Table 3:** Kv1.3 expression on human primary T cell subsets.

T cell subsets	Kv1.3 channels/cell		Kv1.3 channels/pF	
	Resting	Activated	Resting	Activated
CD4 naïve T cell	184.9 ± 41.1	265.2 ± 37.9	143.2 ± 31.9	66.2 ± 8.4
CD4 Tcm cell	324.1 ± 47.2	756.7 ± 65.3	177.2 ± 25.4	279.5 ± 26.7
CD4 Tem cell	246.5 ± 15.6	750.7 ± 57.6	135.8 ± 9.0	261.0 ± 19.4
House dust mite-specific T cell	ND*	1111 ± 151.3	ND*	234.7 ± 23.6

* ND: not determined.

A time course study of Kv1.3 currents in effector memory CD4 T cells showed that Kv1.3 channel numbers increased as soon as 2 hours after activation, reached a peak level at 24 hours and showed a trend to toward decline by 48 hours post-activation (Fig 6D). The dynamics of Kv1.3 expression was different from other T cell activation markers CD69 and CD25 monitored by flow cytometry. CD69 peaked at 10 hours and CD25 increased gradually and was at the highest level at 48 hours after T cell activation. Kv1.3 transcripts in these cell samples were measured by quantitative RTPCR and interestingly the levels were reduced as early as 2 hours after activation, and decreased further overtime to a minimal level 2 days later (Fig 6E). This down-regulation of Kv1.3 transcripts was observed for all T cell subsets including naïve, effector and central memory T cells.

### Kv1.3 blockers effective in blocking antigen-specific T cell response

**Fig 7:**
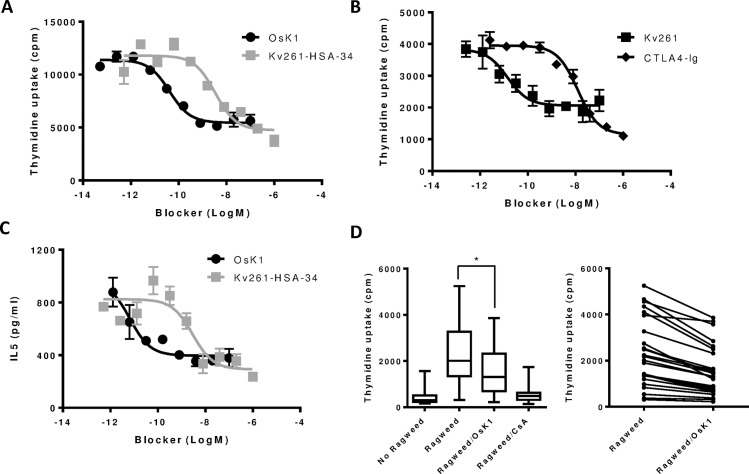
Effect of Kv1.3 blockers on antigen-specific T cell activation. Kv1.3 blocker OsK1, Kv261 peptides and Kv261-HSA-34 fusion protein blocked proliferation of CD4 T cells from house dust mite allergic patients following 5 day stimulation with house dust mite extracts (A, B). IL-5 production from house dust mite-specific T cells was inhibited by Kv1.3 blockers as shown with OsK1 and Kv261-HSA-34 (C). OsK1 peptide at 0.3μM inhibited partially the proliferation of PBMC from patients allergic to ragweed after 3 day stimulation with ragweed extracts (D). Decreased proliferative response of individual donor PBMC samples by OsK1 peptide was shown in a separated graph. CTLA4-Ig or cyclosporine A (1μM) were used as reference compounds. The assays in panels A-C were repeated in 4 independent experiments with samples in duplicates. Data from one representative experiment were presented. The study in panel D was done in an experiment with 29 subjects per group. Statistical significance on the difference in proliferation between untreated and OsK1 treated groups were analyzed by 2-way ANOVA and the P value was determined and indicated as *P < 0.05. Readouts in (A) and (C) were performed on CD4 T cells from the same donor whereas those in (B) were from a separate donor. Data from one representative experiment were presented.

Our results show that the effect of Kv1.3 blockers is highly dependent on the T cell identity and stimulation conditions. In order to better understand the therapeutic potential of Kv1.3 blockers therefore, we investigated the effects of Kv1.3 blockers on T cells activated by disease relevant antigens. A house dust mite-specific T cell recall response could be demonstrated by stimulating PBMC from allergic donors with house dust mite extracts. Kv1.3 blockers including OsK1 and Kv261 peptides, as well as Kv261-HSA-34 partially inhibited T cell proliferation and IL-5 production (Fig 7A–7C). The inhibitory effects of Kv1.3 blockers were shown to be comparable to that of the CD28 blocker CTLA4-Ig, which is a T cell activation blocker that has been shown to be efficacious in the treatment of allergic airway inflammation [40]. Potencies of Kv1.3 blockers on house dust mite-specific response corresponded well with their potencies in blocking Kv1.3 currents, suggesting that the effects were from on-target mechanisms (Fig 7A–7C, Tables 1 & 2). The effect of OsK1 peptide in blocking the allergic specific response was further tested in PBMC purified from allergic rhinitis patients. As shown in Fig 7D, PBMC from 29 patients stimulated with ragweed extracts triggered a profound proliferative response in most of the samples and OsK1 peptide at 0.3μM significantly reduced the ragweed response to about 60% of the induced level. The inhibitory effect of OsK1 peptide was demonstrated in all individual donor samples. In the same assay, the immune suppressor cyclosporine A at 1μM reduced the proliferative response to its basal level.

## Discussion

Kv1.3 is known to play a role in modulating T cell activation [3, 41] and Kv1.3 blockers may have potential therapeutic effect on treatment of autoimmune diseases. In order to better understand this potential however, more information is needed on the activity profile of Kv1.3 blockers across various T cell subsets, and disease relevant T cells, under a range of stimulation conditions.

In our studies, Kv1.3 blockers were only partially efficacious at inhibiting T cell proliferation under various experimental conditions. The effects of Kv1.3 blockers are in clear contrast to the calcineurin inhibitor, cyclosporine A, which fully inhibits T cell activation in all stimulation conditions. The partial efficacy of Kv1.3 inhibitors probably reflects the inability to fully suppress calcium entry. Indeed, T cell activation by thapsigargin, an inhibitor of SERCA that acts exclusively through induction of calcium flux, was only partially inhibited by Kv1.3 blockers. Furthermore, we and others show that calcium fluxes induced by coupling of TCR and CD28 with antibodies, or by thapsigargin treatment, were only partially inhibited by Kv1.3 blockers [42]. During the course of our calcium flux experiments we also found evidence for a population of T cells that were completely insensitive to the effects of Kv1.3 blockers. Since the mechanism of action of Kv1.3 blockers involves depolarization-induced inhibition of calcium flux [41], it is likely that the partial inhibitory effect on calcium entry and the downstream T cell response reflects the involvement of multiple potassium channel sub-types in the maintenance of T cell membrane potential. The residual calcium flux that is resistant to Kv1.3 blocker has previously been shown to be very sensitive to the potassium diffusion potential and chloride- removal, suggesting that other potassium channels distinct from Kv1.3 and perhaps chloride channels can maintain a sufficiently negative membrane potential to drive calcium influx in the absence of Kv1.3 [41, 42].

Further investigation revealed that inhibition of proliferation by Kv1.3 blockers was highly dependent on stimulation strength on both CD8 and CD4 T cells. The mechanism underlying sensitivity to stimulation strength is still unclear. It may reflect involvement of calcium-independent activation pathways in the proliferation response to higher stringency stimulation. Blockers of Kv1.3 have previously been shown to be more effective in suppressing T cell activation induced by signals that elicit a rise in intracellular calcium (e.g., anti-CD3, thapsigargin, ionomycin, antigens, house dust mite) than signals that are independent of calcium flux (e.g., anti-CD28, IL-2) [3, 4, 43–46]. Furthermore, exogenous IL-2 has been shown to over-ride Kv1.3 blocker effect on T cell activation [3, 43]. Increased IL-2 production elicited by stronger stimuli likely contributes to the reduced effectiveness of Kv1.3 blockers. Interestingly, inhibition of IL-2 secretion was relatively independent of stimulus strength possibly suggesting a strong reliance on calcium dependent pathways regardless of the nature of the stimulus.

Kv1.3 blockers have previously been shown to preferentially block the activity of effector memory T cells [5, 6]. In the present study we compared the effects of Kv1.3 blockers side-by-side on primary naïve, central memory and effector memory CD4 T cells. Our findings are largely in agreement with previous studies in that effector memory cells seemed to be most sensitive to the effects of Kv1.3 blockade whereas the efficacy of Kv1.3 blockers was lowest in central memory cells.

Increased functional expression of Kv1.3 has previously been proposed as the basis for the increased sensitivity of effector memory T cells to Kv1.3 blockade, relative to other T cell subsets [5, 7, 32]. In our studies, Kv1.3 currents were elevated in effector memory T cells and antigen-specific CD4 T cells enriched by repeated antigen stimulation, but, in contrast to previous findings, similar increases were also seen in central memory cells. Based on our results, changes in Kv1.3 levels alone cannot explain the differential sensitivity of effector and central memory cells to Kv1.3 blockade. As previously suggested [5], it is possible that changes in expression of other potassium channels, such as KCa3.1, are responsible for the relative insensitivity of central memory cells to Kv1.3 blockade, but KCa3.1 levels were not measured in our study. The reason for the difference between our study and previous studies is not clear. It may reflect differences in the patch clamp method and associated cell selection technique or differences in the methods used to select T cell populations (primary cells versus T cell lines). Previous studies were conducted manually with visual selection of cells from T cell lines whereas the present study adopted an automated approach using primary cells.

Our recordings show that the increase in Kv1.3 currents in effector memory cells occurs rapidly after activation, peaking by 24 h and then declining by day 2. Interestingly, Kv1.3 mRNA measured by quantitative RTPCR was found to decrease rapidly in all T cell subsets after activation. Similar decreases in Kv1.3 mRNA have been described following concanavalin A stimulation of cultured human T cells [47]. These findings suggest that post-transcriptional mechanisms may be responsible for the increased Kv1.3 currents, possibly involving enhanced trafficking of Kv1.3 channels to the plasma membrane [48], or an activation of Kv1.3 channels through protein modification [49–51].The rapid down-regulation of Kv1.3 transcripts following T cell activation may also suggest that Kv1.3 is more important during the early phase of T cell activation, as first suggested by Lin et al. [3].

The partial and variable efficacy of Kv1.3 blockers observed in our studies raised the question as to what effects Kv1.3 blockers might have on disease relevant antigen-specific T cells. All the Kv1.3 blockers used in our studies were shown to partially block house dust mite-specific T cell proliferation and cytokine production with IC_50_ values corresponding to their potencies on Kv1.3 channels. Interestingly, a similar partial effect was also seen with CTLA4-Ig, an FDA-approved T cell inhibitor which blocks co-stimulatory CD28 signals, suggesting that this level of inhibition might be clinically meaningful. OsK1 peptide also partially reduced ragweed induced proliferation in PBMC from 29 ragweed allergic patients. The Kv1.3 blocker, ShK186, has also recently been reported to partially inhibit disease relevant antigen-induced activation of T cells isolated from peripheral blood of patients with asthma [46]. Together, these results suggest that Kv1.3 blockers could be of value to reduce T cell mediated allergic responses.

In summary, the present study provides confirmatory and in some cases, additional information on the profile of activity of Kv1.3 blockers. Our studies reveal that inhibition of T cells by Kv1.3 blockers varies in a complex, multifactorial manner, dependent on the nature of the stimulus, the T cell response and the T cell identity. The profile of activity Kv1.3 blockers is highly differentiated from existing immune-suppressant drugs such as cyclosporine A. Whether such a unique profile of T cell modulation will translate into safer and more effective therapeutics for the treatment of T cell mediated diseases is still unclear and further studies, including clinical studies with potent selective Kv1.3 blockers are required in order to fully understand the potential of this immune-modulatory pathway.

## Supporting Information

S1 Fig Effects of OsK1 and ShK peptides on calcium flux in T cells. OsK1 and ShK peptides (1nM) reduced calcium flux induced by either anti-CD3/CD28 (A) or thapsigargin (B) in human CD4 T cells. Kinetics of calcium flux in different samples is presented as curves over time (A, B). CD4 T cell populations with different levels of calcium signals are presented in dot plots and their Fluo4 intensities in geometric means as well as population percentages are indicated (C).
(TIF)
