# Supplementary figures and images for: T Cell Subset and Stimulation Strength-Dependent Modulation of T Cell Activation by Kv1.3 Blockers
Source: PLoS One. 2017 Jan 20;12(1):e0170102. doi: 10.1371/journal.pone.0170102 (PMC5249144; doi:10.1371/journal.pone.0170102)

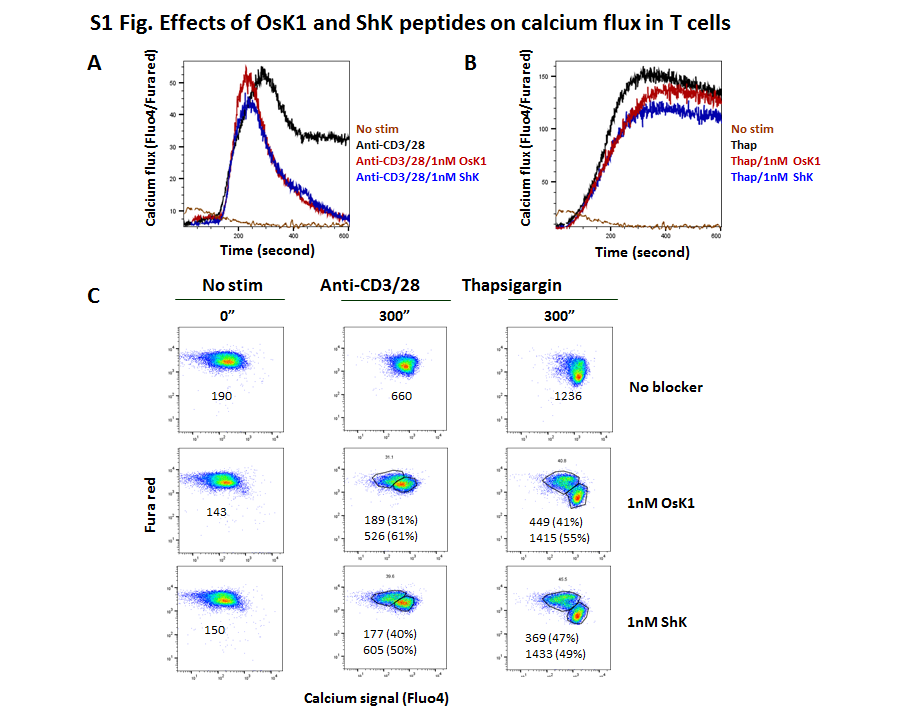

Supplement: S1 Fig — OsK1 and ShK peptides (1nM) reduced calcium flux induced by either anti-CD3/CD28 (A) or thapsigargin (B) in human CD4 T cells. Kinetics of calcium flux in different samples is presented as curves over time (A, B). CD4 T cell populations with different levels of calcium signals are presented in dot plots and their Fluo4 intensities in geometric means as well as population percentages are indicated (C). (TIF) [file pone.0170102.s001.tif]
